# Widespread choroid plexus contamination in sampling and profiling of brain tissue

**DOI:** 10.1038/s41380-021-01416-3

**Published:** 2022-01-05

**Authors:** Kimberly C. Olney, Kennedi T. Todd, Praveen N. Pallegar, Tanner D. Jensen, Mika P. Cadiz, Katelin A. Gibson, Joseph H. Barnett, Camila de Ávila, Samantha M. Bouchal, Benjamin E. Rabichow, Zonghui Ding, Aleksandra M. Wojtas, Melissa A. Wilson, John D. Fryer

**Affiliations:** 1 Department of Neuroscience, Mayo Clinic, Scottsdale, AZ 85259 USA; 2 School of Life Sciences, Arizona State University, Tempe, AZ 85282 USA; 3 Center for Evolution and Medicine, Arizona State University, Tempe, AZ 85282 USA; 4 Mayo Clinic MD/PhD Training Program, Scottsdale, AZ 85259 USA; 5 Mayo Clinic Graduate School of Biomedical Sciences, Scottsdale, AZ 85259 USA; 6 The Biodesign Center for Mechanisms of Evolution, Arizona State University, Tempe, AZ 85282 USA

**Keywords:** Neuroscience, Diseases

## Abstract

The choroid plexus, a tissue responsible for producing cerebrospinal fluid, is found predominantly in the lateral and fourth ventricles of the brain. This highly vascularized and ciliated tissue is made up of specialized epithelial cells and capillary networks surrounded by connective tissue. Given the complex structure of the choroid plexus, this can potentially result in contamination during routine tissue dissection. Bulk and single-cell RNA sequencing studies, as well as genome-wide in situ hybridization experiments (Allen Brain Atlas), have identified several canonical markers of choroid plexus such as *Ttr*, *Folr1*, and *Prlr*. We used the *Ttr* gene as a marker to query the Gene Expression Omnibus database for transcriptome studies of brain tissue and identified at least some level of likely choroid contamination in numerous studies that could have potentially confounded data analysis and interpretation. We also analyzed transcriptomic datasets from human samples from Allen Brain Atlas and the Genotype-Tissue Expression (GTEx) database and found abundant choroid contamination, with regions in closer proximity to choroid more likely to be impacted such as hippocampus, cervical spinal cord, substantia nigra, hypothalamus, and amygdala. In addition, analysis of both the Allen Brain Atlas and GTEx datasets for differentially expressed genes between likely “high contamination” and “low contamination” groups revealed a clear enrichment of choroid plexus marker genes and gene ontology pathways characteristic of these ciliated choroid cells. Inclusion of these contaminated samples could result in biological misinterpretation or simply add to the statistical noise and mask true effects. We cannot assert that *Ttr* or other genes/proteins queried in targeted assays are artifacts from choroid contamination as some of these differentials may be due to true biological effects. However, for studies that have an unequal distribution of choroid contamination among groups, investigators may wish to remove contaminated samples from analyses or incorporate choroid marker gene expression into their statistical modeling. In addition, we suggest that a simple RT-qPCR or western blot for choroid markers would mitigate unintended choroid contamination for any experiment, but particularly for samples intended for more costly omic profiling. This study highlights an unexpected problem for neuroscientists, but it is also quite possible that unintended contamination of adjacent structures occurs during dissections for other tissues but has not been widely recognized.

## Introduction

The mammalian brain is tightly packed with subregions that have unique gene expression profiles. In order to understand these regional differences in brain tissue, scientists often dissect freshly obtained brains into separate regions (e.g., hippocampus, cortex, striatum, thalamus, brain stem, etc.) that are then used to purify RNA, proteins, lipids, or metabolites for further analyses. Often these inquiries utilize targeted methods such as western blotting, RT-qPCR, etc., but frequently investigators choose to perform unbiased “omic” profiling using microarrays, RNA sequencing (RNAseq), proteomics, lipidomics, metabolomics, etc. When analyzing our unpublished datasets, we noticed an occasional differential expression of the transthyretin (*Ttr*) gene between groups that we could not validate, nor did it make biological sense. We decided to examine the expression of transthyretin (*Ttr*) and other similar genes more comprehensively. *Ttr* showed almost exclusive expression in the choroid plexus of the mouse in the Allen Brain Atlas, a unique and powerful resource to examine mRNA levels of virtually every transcript across the brain and visualize the expression from in situ hybridization [1–3]. We also used DropViz.org, a single-cell database collected from nine brain regions of adult mice that is comprised of 690,000 cells [4], and found that *Ttr* was the canonical marker gene that defined choroid plexus cell clusters, with extremely low expression in other cell types. In addition, a comprehensive query of deposited microarray and RNAseq datasets from Gene Expression Omnibus (GEO) profiles [5] suggested that the majority of datasets have at least some unintended choroid contamination, with studies that contain some potential artifacts of expression due to uneven (and unlucky) distribution of choroid contamination. This unfortunate tainting of samples is not unique to mouse datasets, as we also identified likely choroid contamination when examining human datasets deposited in the Allen Brain Atlas as well as the widely used human Genotype-Tissue Expression (GTEx) project and binning of likely “high contamination” vs “low contamination” *TTR* samples yielded high enrichment of choroid plexus marker genes. These findings provide a cautionary tale for neuroscientists and indicate that determining the level of choroid contamination is necessary for proper biological interpretation, especially when factoring in the considerable costs of most omics studies. Examination of other tissues may also reveal other unintended dissection artifacts that are worth reexamination.

## Methods

### GEO query to identify *TTR/Ttr* contamination in human and mouse brain gene expression datasets

The GEO database [5] was used to determine the presence of transthyretin (*TTR, Ttr*) expression in brain tissue datasets from *Homo sapiens* and *Mus musculus*. Our query searched for “Ttr AND (brain OR pons OR medulla OR cortex OR cortical OR hippocampus OR nervous OR cerebellum OR hypothalamus OR thalamus OR striatum OR amygdala OR forebrain)” in the central nervous system (CNS) of *Homo sapiens* and *Mus musculus*. This yielded 586 GEO Profiles. Each sample within each profile has a value and rank for *TTR*/*Ttr* expression. Values stem from the original user-supplied data that may be, but not limited to, raw counts, log-transformed counts, or fold change [5]. *TTR/Ttr* value for each sample is displayed with a red bar. Ranks are determined by placing all gene expression values per sample in bins from 0 to 100 and then assigning a percentile to each gene. Ranks are denoted with blue squares [5].

The query of 586 GEO profiles was first filtered to exclude studies where group-level differences were likely due to intrinsic biology (e.g., lung vs brain), studies on cultured cells, predominantly tumor profiling, and studies listed more than once in the query results (e.g., more than one microarray probe identifier). GEO profiles that contained faded-out bars or squares to signify absent calls with Affymetrix microarrays were included. Post-filtering yielded 158 GEO profiles (Supplementary Table 1). Of the 158 GEO profiles, 23 are datasets from *Homo sapiens* and 135 are *Mus musculus*. After compiling the data from GEO profiles into dictionaries of profiles and their corresponding ranks, each profile was assigned 1 of 5 priority numbers to categorize the level of *TTR/Ttr* contamination (Table 1). A one-way ANOVA test was run on each of the 158 profiles that assessed the *TTR/Ttr* rank across experimental groups and a Bonferroni correction was applied to the *p* values. Fifteen of the GEO profiles produced NA values during the ANOVA test and thus we removed from downstream analysis, 143 profiles remained. If the one-way ANOVA test returned an adjusted *p* value less than 0.05, this was given a rank priority score of 1, denoting a strong trend in differential *TTR/Ttr* levels between experimental groups (Table 1 and Supplementary Table 1). If the one-way ANOVA test returned an adjusted *p* value between 0.05 and 0.1, this was given a rank priority score of 2, denoting a moderate trend in *TTR/Ttr* levels. Profiles with a non-uniform distribution of *TTR/Ttr* expression and no group-level trends were assigned a rank priority number of 3. Those with a uniformly high rank of *TTR/Ttr* across experimental groups, with at least 80% of samples with a rank in the 80th percentile or more, were classified as rank priority number 4. Finally, a rank priority number of 5 was given to profiles with a uniformly low rank *TTR/Ttr* expression across experimental groups, with at least 80% of samples with *TTR/Ttr* rank in the 20th percentile or lower (Table 1). We wanted to investigate if this possible choroid contamination was evident in other species datasets, and thus did a manual search to find other species datasets that may have unintended group-level differences for *TTR* expression.

**Table 1:** Rank priority numbers for *TTR* or *Ttr* contamination or lack of contamination among samples within a GEO dataset.

Rank priority (blue squares)
1 = *TTR/Ttr* expression almost exclusively between groups, one-way ANOVA test *p* value < 0.05
2 = *TTR/Ttr* expression moderately between groups, one-way ANOVA test *p* value < 0.1 and ≥0.05
3 = *TTR/Ttr* expressed among samples with no clear pattern (random)
4 = *TTR/Ttr* expressed highly in most samples (rank or value ≥80%, in >80% of samples)
5 = *TTR/Ttr* expressed lowly in most samples (rank or value ≤20%, in >80% of samples)

### Quantifying *TTR* expression in human brain regions

We investigated how potential *TTR* contamination may alter biological interpretation using human brain data from the Allen Brain Atlas [1]. The human Allen Brain Atlas is a publicly available gene expression resource comprising multiple datasets from various genome-wide microarray or RNAseq-based projects that additionally include histologic data [1, 2]. Specifically, we downloaded the aging, dementia, and traumatic brain injury (TBI) RNAseq expression data that include 377 samples from hippocampus, parietal cortex, temporal cortex, and frontal white matter from 107 individuals [2]. There are 94 hippocampus, 91 parietal cortex, 99 temporal cortex, and 93 frontal white matter samples in this dataset [2]. The un-normalized gene-level transcripts per million (TPM) values were downloaded from Allen Brain Atlas for each brain region (https://aging.brain-map.org/data/tbi_data_files.csv), as well as de-identified clinical information for all donors within the study [1, 2]. There are 50,283 genes in the Allen Brain Atlas data; we removed MT genes as these genes are highly expressed and could skew our results. After removing MT genes, there are 50,246 genes remaining. Each gene was assigned a percentile rank. This was accomplished by placing all gene expression values per sample in bins from 0 to 100 and then assigning a percentile to each gene. We then examined the percentile rank of *TTR* for each sample in each brain region. Then, to determine if potential choroid plexus contamination could contribute to biological misinterpretation, we ran a differential expression analysis using the Allen Brain Atlas hippocampus samples as this region is in close proximity to the choroid plexus.

Examining the 94 hippocampus samples from the Allen Brain Atlas data, we assigned each sample as either having little choroid plexus contamination or as potentially having a lot of choroid plexus contamination. To assign the samples to a group, we plotted the log_2_ (TPM) of *TTR* and defined samples with a log_2_(*TTR*) of less than 3.32 as having low choroid plexus contamination and samples with a log_2_(*TTR*) greater than 5.32 as potentially having high choroid plexus contamination. Samples with greater than 3.32 log_2_(*TTR*) expression, but less than 5.32 log_2_(*TTR*) expression were not assigned to either the low or high contamination groups. There are 58 samples in the low contamination group and 10 samples in the potentially high contaminated group, and 26 samples in neither low nor high contamination groups. The TPM value data for all genes were then converted to counts per million (CPM), and then filtered to keep genes with greater than 2 CPM in at least three samples. Post-filtering resulted in 21,959 genes. The counts data for each sample were then normalized for gene expression distributions by calculating the trimmed mean of M-values with edgeR [6]. To normalize expression intensities, a weight for each observation was generated as part of the voom method [7]. The Allen Brain Atlas human data are comprised of both male and female samples that range in age of 77–100+ years old, as such, both sex and age were included as covariates in the differential expression model. Allen Brain Atlas donor age is denoted in bins of every 5 years. A non-parametric Kruskal–Wallis rank-sum test was performed to determine differences in log_2_(*TTR*) expression distributions among the age bins for Allen Brain Atlas [8]. Differential expression analyses between the low and high contamination groups were then carried out by linear modeling as implemented in the R package limma [9]. Genes are defined as being differentially expressed if the adjusted *p* value is <0.05 and absolute log_2_ fold change >1 (Supplementary Table 2). The above differential expression analysis was repeated for each sex independently to quantify the degree of variation in the low and high contamination groups within each sex. We also employed a differential expression analysis between the low and high contamination groups using the choroid plexus marker folate receptor 1 (*FOLR1*) (Supplementary Table 2). Re-examining the 94 hippocampus samples from the Allen Brain Atlas data, we assigned each sample as either having little choroid plexus contamination or as potentially having a lot of choroid plexus contamination. To assign the samples to a group, we plotted the log_2_(TPM) of *FOLR1* and defined samples with a log_2_(*FOLR1*) of less than −1 as having low choroid plexus contamination and samples with a log_2_(*FOLR1*) greater than 1 as potentially having high choroid plexus contamination. There are 40 samples in the low contamination group and 3 samples in the potentially high contaminated group. The remaining 51 samples were not assigned to a group and were not included in the *FOLR1* differential expression analysis.

We additionally investigated choroid plexus expression in GTEx brain samples. The GTEx project provides open access RNAseq expression data from 54 non-diseased tissue sites across nearly 1000 individuals [10]. We downloaded the gene TPM expression data GTEx_Analysis_2017-06-05_v8_RNASeQCv1.1.9_gene_tpm.gct.gz as well as de-identified sample attributes (https://gtexportal.org/home/datasets). There are 12 brain regions in the GTEx data (Supplementary Table 3). The TPM expression data for each brain region includes counts information for 54,592 unique genes. After removing MT genes, there are 54,555 genes remaining. Lowly contaminated and potentially highly contaminated samples were defined using the same thresholds as described in the Allen Brain Atlas analysis above. There are 34 likely lowly contaminated and 100 potentially highly contaminated GTEx hippocampus samples. The remaining 63 samples were not assigned to either the low or high contamination groups and were not included in the downstream analysis. The low and high contamination samples were analyzed in the same manner as the Allen Brain Atlas data. After filtering lowly expressed genes, 24,330 genes remained for downstream analysis. Differential gene expression was performed utilizing the exact same tools and parameters as described in the Allen Brain Atlas hippocampus methods above. The GTEx data are composed of both male and female samples that range in age from 20 to 79 years old, with age denoted in bins every 10 years. Both sex and age were included as covariates in the differential expression model. In addition, a non-parametric Kruskal–Wallis rank-sum test was performed to determine differences in the log_2_(*TTR*) expression distributions among the age bins. Differential expression analyses between the lowly and highly contaminated groups were then carried out by linear modeling as implemented in the R package limma [9]. Genes are defined as being differentially expressed if the adjusted *p* value is <0.05 and absolute log_2_ fold change >1 (Supplementary Table 4). The above differential expression analysis was repeated for each sex independently to quantify the degree of variation in the low and high contamination groups within each sex. Finally, we employed a differential expression analysis between the low and high contamination using the choroid plexus marker *FOLR1*. For the reexamination between low and high contamination samples using the *FOLR1* marker, we employed the same thresholds for defining contamination groups as described in the Allen Brain Atlas methods above for *FOLR1*. The GTEx data used for the analyses described in this manuscript were obtained from the GTEx Portal on January 6, 2021.

Data processing pipeline available on GitHub (https://github.com/olneykimberly/TTR).

## Results

### TTR, FOLR1, and PRLR are markers of the choroid plexus

**Fig. 1:**
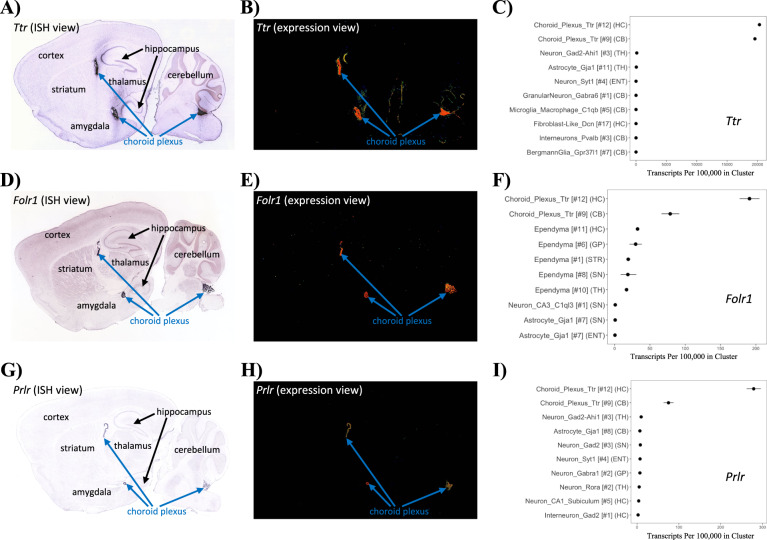
*Ttr*, *Folr1*, and *Prlr* are markers of the choroid plexus. **A** Allen Mouse Brain Atlas in situ hybridization of *Ttr* shows presence largely in the choroid plexus; image credit Allen Institute. **B** Utilizing the background-subtracted “expression view” demonstrates *Ttr* expression predominantly in the choroid plexus. **C** To confirm the findings from the Allen Mouse Brain Atlas exploration, we searched *Ttr* on DropViz.org, which shows *Ttr* to be highly expressed in choroid plexus cell-type clusters #12 and #9 with greater than 15,000 transcripts per 100,000 in clusters. The above inquiry was repeated for *Folr1* (**D**–**F**) and *Prlr* (**G**–**I**), which confirms that these genes *Ttr, Folr1*, and *Prlr* are predominantly expressed in the choroid plexus. The DropViz.org plots **C**, **F**, and **I** show the regions across the brain in order from greatest to least expression for the top ten regions.

This current study was born of frustration from our occasional inability to validate some of our own unpublished RNAseq experiments that identified *Ttr* or other genes that were putatively differentially expressed. To examine this further, we used the available in situ hybridization images from Allen Brain Atlas [3] and found that *Ttr* is almost exclusively expressed in the choroid plexus as evidenced from the raw in situ hybridization images (Fig. 1A) as well as the background-subtracted “expression view” images (Fig. 1B). To better understand *Ttr* expression in the brain using a complementary approach, we next searched *Ttr* on DropViz.org [4], a comprehensive single-cell RNAseq database of the adult mouse brain, and found *Ttr* to be highly and almost exclusively expressed in choroid plexus cell clusters with very low expression in other cell types (Fig. 1C). In fact, *Ttr* was used to define the choroid plexus in their clustering analysis. Using the cluster explorer function in DropViz.org, we also identified several other genes with highly selective expression in choroid such as *Folr1* and prolactin receptor, *Prlr*. We visualized the expression of these genes in Allen Brain Atlas and found a nearly exclusive expression within the choroid plexus in the in situ hybridization and expression view images (Fig. 1D–H). We confirmed that the expression of these genes was highly selective in choroid plexus cells by downloading and replotting data from DropViz.org (Fig. 1F, I). Although their expression is not zero in other brain cell types, *Ttr*, *Folr1*, and *Prlr* are reasonably strong markers of the choroid plexus with *Ttr* being the most specific.

### Survey of GEO database reveals potential unintended group-level differences for *TTR* expression

**Fig. 2:**
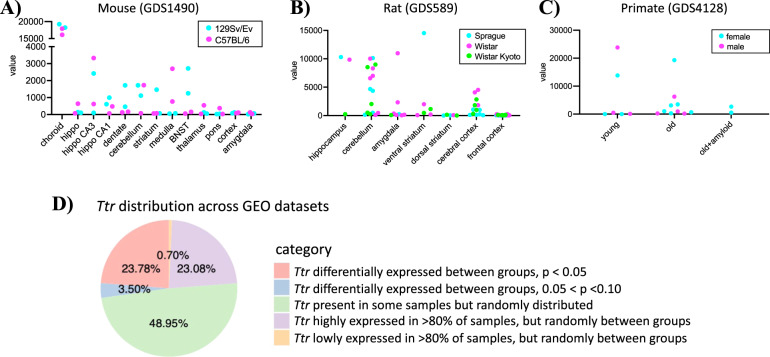
*Ttr *expression in GEO profiles reveals potential between-group contamination biases. **A** *Ttr* expression in several *Mus musculus* brain regions from 129SV/EV (teal) and C57BL/6 (magenta) lines from GEO profile GDS1490. Each point represents a sample. The GDS1490 study shows *Ttr* to be highly expressed in the choroid plexus tissue, followed by the hippocampus and the cerebellum. **B** GEO dataset GDS589 of rat brain regions from Sprague Dawley (teal), Wistar (magenta), and Wistar Kyoto (lime) strains shows a wide range of *Ttr* expression in numerous brain regions, with the highest observed in the ventral striatum. Each point represents a sample. Sprague Dawley and Wistar rats have >40× higher expression of *Ttr* in the hippocampus compared to Wistar Kyoto rats. **C** *Ttr* expression in the temporal cortex from the gray mouse lemur (*Microcebus murinus*) from GEO dataset GDS4128 shows an enormous range in expression among samples with a young female (teal) and a male (magenta) sample showing several orders higher expression for *Ttr* compared to the other young male and female samples within the same group. Each point on the plot represents a sample. Finally, **D** *Ttr* distribution across 143 GEO profiles shows between-group *TTR/Ttr* expression differences and that the majority of the studies surveyed show within-dataset expression variability for *TTR/Ttr* expression.

Under this assumption, we conducted a query looking at *TTR*/*Ttr* expression in the CNS of profiling datasets to investigate the potential scope of choroid plexus contamination in a wide variety of studies and samples. We used the GEO repository for our query as GEO houses thousands of gene expression data experiments [5]. To be as inclusive as possible, we used the search term “Ttr AND (brain OR pons OR medulla OR cortex OR cortical OR hippocampus OR nervous OR cerebellum OR hypothalamus OR thalamus OR striatum OR amygdala OR forebrain)” to query GEO Profiles (https://www.ncbi.nlm.nih.gov/geoprofiles) (Supplementary Table 1). Perusing these GEO profiles revealed numerous and striking examples of widely variable *Ttr* expression that occurred in multiple species. To illustrate these examples, we replotted *Ttr* expression from a subset of samples from these datasets. The GEO profile GDS1490 contains 24 neural tissues and 10 body regions from adult male mice of mice [11]. We focused on the brain samples in this dataset and found that *Ttr* has the highest expression in the choroid plexus samples as would be expected of this marker gene, but several other brain regions had highly variable *Ttr* expression with individual samples showing differences across several orders of magnitude (Fig. 2A). Of note, samples in closer physical proximity to the choroid were most likely to suffer from this highly variable choroid contamination, including the hippocampus and CA1/CA3 subregions as well as the cerebellum, bed nucleus of the stria terminalis, medulla, and even the striatum. Compared to 129Sv/Ev mice, it is possible that C57BL/6 mice have higher expression of *Ttr* in the medulla or lower *Ttr* expression in the bed nucleus of the stria terminalis (Fig. 2A), but a more plausible explanation for these extraordinarily high levels is that there was an unlucky inclusion of choroid tissue during dissection for some samples but not others. This is a likely occurrence given the vascular and connective tissue that is characteristic of the choroid plexus.

The choroid contamination is not limited to mice, as we identified highly variable *Ttr* expression in datasets from other species. An examination of the GEO dataset GDS589 of rat brain regions from Sprague Dawley, Wistar, and Wistar Kyoto strains [12] again showed a widely variable outlier expression of *Ttr* in numerous brain regions (Fig. 2B). Again, it is biologically possible that Sprague Dawley and Wistar rats have >40× higher expression of *Ttr* in the hippocampus compared to Wistar Kyoto rats, but the more likely explanation is that these samples had choroid carryover from dissection. Similar examples are seen in the cerebellum, amygdala, striatum, and even cortex from this dataset (Fig. 2B). We also examined the expression of *Ttr* in a targeted profiling study of the temporal cortex from the gray mouse lemur (*Microcebus murinus*), a small mammal in the primate family with a gyrencephalic brain, from GEO dataset GDS4128 [13]. The GEO dataset GDS4128 contains 18 samples from both young and old mouse lemurs (male and female) to model cerebral aging and Alzheimer’s disease (AD) [13]. Although this was only analyzed for temporal cortex, we again found that *Ttr* was expressed at several orders of magnitude higher only in a subset of samples that is best explained by choroid contamination rather than biological variation (Fig. 2C).

We were curious to know the scope of potential choroid plexus contamination among these GEO profiles and categorized them based on whether *Ttr* was abundant and whether it was evenly or unevenly distributed among experimental groups. We downloaded expression data from 143 GEO profiles and used a one-way ANOVA to compare *TTR/Ttr* rank levels among groups and corrected for multiple testing applying the Bonferroni correction. Of the 143 GEO profiles, 21 are datasets from *Homo sapiens* and 122 are *Mus musculus* datasets. To our surprise, we found *TTR/Ttr* rank to be differentially expressed between groups in 23.78% of studies (adjusted *p* value < 0.05), which could potentially bias the biological interpretation (Fig. 2D). An additional 3.5% of the studies showed moderate group-level differences in *TTR/Ttr* expression (adjusted *p* value > 0.05 and <0.1) (Fig. 2D). Thus, over a quarter of these samples had a reasonably strong group-wise selective contamination from choroid. We admit that this is not the appropriate statistical test to apply for transcriptomic studies, but we used it as a way to categorize those samples that may have suffered from a skewed choroid contamination between groups.

*TTR/Ttr* was also found to be highly expressed but with no clear group-level differences in an additional 27.2% of these 143 GEO profiles (Fig. 2D). In an additional 33.5% of the studies, *TTR/Ttr* was present but showed random expression among samples with no clear group-level differences. Remarkably, only a single human study, 0.6% of the GEO profiles, appeared to have *TTR* percentile rank below the 20% percentile in at least 80% of the samples within the study (Fig. 2D). This GEO Profiles inquiry was not meant to cast doubt on previous experiments, but rather to highlight the apparent choroid contamination across a wide variety of studies. Whether these are true biological differences in *TTR/Ttr* expression or due to choroid contamination is up to each individual researcher to reexamination these datasets.

### Regions in close proximity to the choroid plexus are more likely to be contaminated

**Fig. 3:**
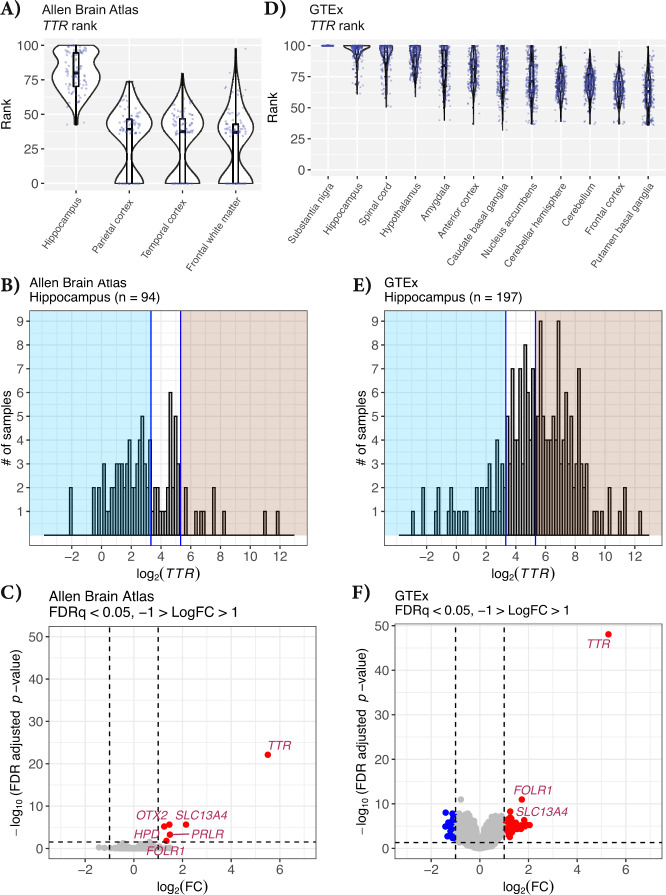
*TTR* percentile rank for regions in the human brain. Violin jitter of TTR percentile rank for each brain region in: **A** The Allen Brain Atlas data and **B** GTEx. The highest mean *TTR* percentile rank in the Allen Brain Atlas data is observed in the hippocampus. The highest mean *TTR* percentile rank observed in the GTEx data is substantia nigra and hippocampus. **C** Histogram of the log_2_(*TTR)* expression among hippocampus samples within the Allen Brain Atlas data and **D** GTEx. The left blue-shaded regions indicate the samples that likely do not have choroid plexus contamination, log_2_(*TTR*) < 3.32. The right red-shaded region indicates samples that potentially have choroid plexus contamination, log_2_(*TTR*) > 5.32. **E** Volcano plot showing the gene differential expression between the potentially contaminated and likely not contaminated Allen Brain Atlas hippocampus samples (*n* = 68) and **F** GTEx hippocampus samples (*n* = 97). Vertical lines show the log_2_ fold change of 1 and −1. Horizontal line indicates the adjusted *p* value cutoff of 0.05. Six genes are upregulated in the potentially contaminated group, adjusted *p* value < 0.05 and absolute log_2_ fold change > 1. Each point represents a gene, points in blue are downregulated, FDRq < 0.05 and the log_2_ fold change less than −1. Upregulated genes are indicated in red, FDRq < 0.05 and the log_2_ fold change greater than 1. Genes that are not differentially expressed are shown in gray.

The in situ hybridization images of mouse tissue from the Allen Brain Atlas and data shown in Figs. 1 and 2 indicate that regions near the choroid plexus such as the hippocampus would be more likely to have sample contamination from *Ttr* and other choroid transcripts. We next sought out independent sources of deposited data to further understand the potential contribution of this choroid contamination. We first downloaded the expression values of *TTR* from the human aging, dementia, and TBI dataset in Allen Brain Atlas [1, 2]. In this data, the hippocampus shows the most variable and highest mean *TTR* percentile rank among samples compared to other brain regions (Fig. 3A). The mean *TTR* percentile rank for the hippocampus is 80.12 in the Allen Brain Atlas data, with several samples at or near the 100th percentile (Fig. 3A). In contrast, the parietal cortex, temporal cortex, and frontal white matter show *TTR* in lower percentile ranks at a mean of 32.22, 28.63, and 27.65, respectively (Fig. 3A). The lowest sample percentile rank of *TTR* for the hippocampus is 42.6, and the highest being 99.96. Like the hippocampus samples, we observe a wide range in the *TTR* percentile rank among frontal white matter samples, with some samples showing *TTR* in the 0 percentile rank, while other samples show *TTR* in the 97.54 percentile (Fig. 3A). This wide range of *TTR* expression percentile rank among samples suggests that some samples may have been inadvertently contaminated with choroid plexus during tissue dissection. To exclude the possibility of biological differences of *TTR* expression from aging and dementia samples from Allen Brain Atlas, we repeated this analysis, examining *TTR* percentile rank expression in the GTEx dataset [10], focusing only on the 12 brain regions that were profiled. The GTEx consortium comprises non-diseased tissues from donors ranging in age from 20 to 79 years old. Like the Allen Brain Atlas data, we observe that tissues closest to the choroid plexus show the highest mean *TTR* percentile rank expression (Fig. 3B). Substantia nigra and the hippocampus show the highest mean *TTR* percentile rank out of the 12 GTEx brain regions, with a mean percentile rank of 100 and 94.70, respectively (Fig. 3B). The regions with the lowest mean percentile rank of *TTR* are the basal ganglia (putamen) and the frontal cortex, at a mean percentile rank of 62.70 and 65.11, respectively (Fig. 3B). Similar to the Allen Brain Atlas data, we observe a wide range in the percentile rank of *TTR* among samples for a given tissue. The lowest observed percentile rank of *TTR* in the basal ganglia (caudate) is 32, while the highest within that tissue is 100. Unlike the Allen Brain Atlas data, there are no samples with a *TTR* percentile rank below 32 in GTEx, whereas some Allen Brain Atlas samples show *TTR* in the zero percentile rank. Regions nearest the choroid plexus show the highest mean percentile rank of *TTR*, and there are wide ranges in *TTR* percentile rank expression among samples within tissues (Fig. 3A, B). As highlighted above, some of these might be driven by true biological differences, but the most parsimonious explanation is that many of these instances simply had contamination from the choroid plexus.

### TTR expression is a strong indicator of choroid contamination

We next asked whether a differential expression analysis of “low” vs “high” *TTR* expressing samples might provide additional support for our choroid contamination hypothesis. We focused on the hippocampus and plotted the expression values for *TTR* in both the Allen Brain Atlas dataset (Fig. 3B) and GTEx dataset (Fig. 3E). Note that X-axis is in log_2_ and further highlights that some samples vary in *TTR* expression across several orders of magnitude (Fig. 3B, E). There is no clear bimodal distribution of *TTR* expression in either the Allen Brain Atlas or GTEx datasets (Fig. 3B, E). In order to have a sufficient number of samples in each low or high contamination group, we visually selected a log_2_(*TTR)* expression of 3.32 to define lowly contaminated samples and log_2_(*TTR*) of 5.32 to define potentially highly contaminated samples. These values retained enough samples in each group for each dataset. Using an arbitrary delineation of “low” (blue shading) vs “high” (red shading) *TTR* expression, we found several differentially expressed genes between these groups for both the Allen Brain Atlas dataset (Fig. 3C) and the GTEx dataset (Fig. 3F), with many more genes present in the latter due to higher sample numbers and therefore greater statistical power (also see Supplementary Tables 2 and 4). Among the most enriched genes for both datasets are several choroid plexus marker genes, including *FOLR1*. Gene ontology analysis of the 69 “upregulated” genes observed in the GTEx analysis showed highly significant enrichment for axoneme assembly, cilium movement involved in cell motility, ensheathment of neurons, motile cilium assembly, and transport of small molecules (Supplementary Fig. 1), all clear characteristics of the choroid plexus. Finding such a high enrichment of choroid marker genes by comparing high vs low *TTR* groups is most likely explained by unequal distribution of choroid contamination.

We also investigated *TTR* expression distribution among the age bins in Allen Brain Atlas and GTEx data to determine age differences for *TTR* expression. The Allen Brain Atlas donors range from 75 to 100+ years old and are denoted in bins every 5 years. The GTEx donors range from 20 to 79 years old and are denoted in bins every 10 years. Utilizing a non-parametric Kruskal–Wallis rank-sum, we determined differences in log_2_(*TTR)* expression distributions among the age bins and found no statistically significant difference between *TTR* expression by different age bins for the Allen Brain Atlas data (*H* = 87.66, df = 91, *p* value = 0.58) or for the GTEx data (*H* = 196, df = 196, *p* value = 0.49) (Supplementary Fig. 2).

### TTR is upregulated in the high contamination group regardless of dataset or genetic sex

Running the same differential expression analysis described above for each sex independently in both the Allen Brain Atlas and GTEx datasets, we observe that *TTR* continues to be upregulated in the high contamination group, regardless of dataset or genetic sex (Supplementary Figs. 3 and 4 and Supplementary Tables 2 and 4). There are 41 female hippocampus samples in the Allen Brain Atlas dataset; 23 show low contamination, and 5 show high contamination (Supplementary Fig. 3A). For the male Allen Brain Atlas hippocampus samples, there are 53 donors, 35 of whom show low contamination and 5 show high contamination (Supplementary Fig. 3B). Although underpowered, we still observe *TTR* to be upregulated in the high contamination samples compared to the low contamination samples in both the female-only and male-only analysis, FRDq < 0.05 and log_2_(FC) < 1 (Supplementary Fig. 3C, D). In addition, in the Allen Brain Atlas male-only analysis, we also observe *PRLR*, another choroid plexus marker, to be significantly upregulated in the high contamination compared to the low contamination group. For the GTEx dataset, there are 54 female and 143 male samples. There are 11 low contamination and 32 high contamination samples when looking only at the female donor samples (Supplementary Fig. 4A). We observe 23 low contamination and 68 high contamination samples for the male samples (Supplementary Fig. 4B). Again, we observe that *TTR* is upregulated in the high contamination group, regardless of sex (Supplementary Fig. 4C, D). *FOLR1*, another choroid plexus marker, is also upregulated in the high contamination group regardless of sex (Supplementary Fig. 4C, D). Finding such high enrichment of choroid markers by comparing high vs. low *TTR* groups cannot be explained by sex differences and, instead, are most likely explained by choroid contamination.

### *FOLR1* expression is an indicator of choroid contamination

**Fig. 4:**
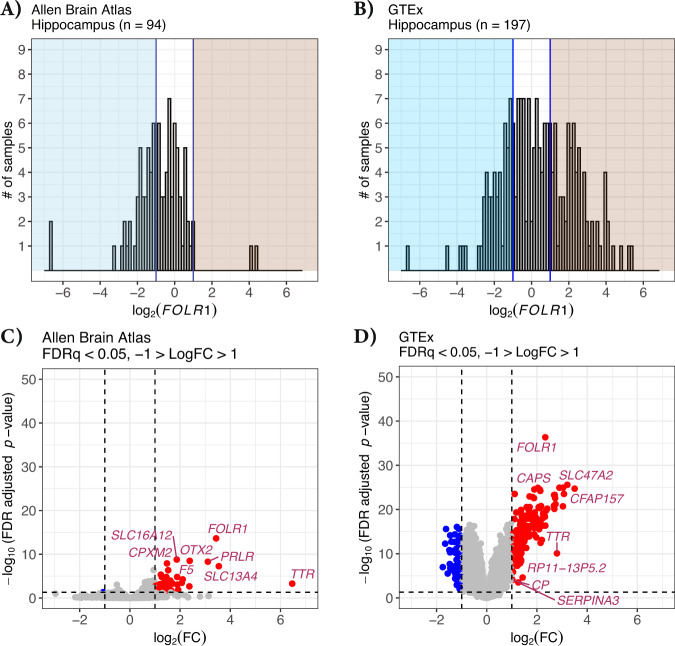
*FOLR1* differential expression between high and low contamination Allen Brain Atlas and GTEx hippocampus samples. **A** Histogram of the log_2_(*FOLR1*) expression among hippocampus samples within the Allen Brain Atlas and **B** GTEx. The left blue-shaded regions indicate the samples that likely do not have choroid plexus contamination, log_2_(*FOLR1*) < −1. The right red-shaded region indicates samples that potentially have choroid plexus contamination, log_2_(*FOLR1*) > 1. **C** Volcano plot showing the gene differential expression between the potentially contaminated and likely not contaminated Allen Brain Atlas hippocampus samples (*n* = 43) and **D** GTEx hippocampus samples (*n* = 123). Vertical lines show the log_2_ fold change of 1 and −1. Horizontal line indicates the adjusted *p* value cutoff of 0.05. Each point represents a gene, points in blue are downregulated, FDRq < 0.05 and the log_2_ fold change less than −1. Upregulated genes are indicated in red, FDRq < 0.05 and the log_2_ fold change greater than 1. Genes that are not differentially expressed are shown in gray.

We next asked whether a different choroid plexus marker might further provide evidence for our choroid contamination hypothesis by examining a differential expression analysis of “low” vs “high” expressing samples using the choroid plexus marker *FOLR1*. Again, we focused on the hippocampus and plotted the expression values for log_2_(*FOLR1*) in both the Allen Brain Atlas dataset (Fig. 4A) and GTEx dataset (Fig. 4B). The X-axis is in log_2_, and like *TTR*, we observe that some samples vary in *FOLR1* expression across several orders of magnitude (Fig. 4A, B). Using an delineation of “low” (blue shading) of log_2_(*FOLR1*) < −1 vs “high” (red shading) of log_2_(*FOLR1*) > 1 expression, we found several differentially expressed genes between these groups for both the Allen Brain Atlas dataset (Fig. 4C) and the GTEx dataset (Fig. 4D), with many more genes present in the latter due to higher sample numbers and therefore greater statistical power (also see Supplementary Tables 2 and 4). Among the most enriched genes for both datasets are several choroid plexus marker genes, including *TTR* and *FOLR1*. Gene ontology analysis of the 178 “upregulated” genes observed in the GTEx analysis showed highly significant enrichment for cilium movement, axonemal dynein complex assembly, and cerebrospinal fluid circulations (Supplementary Fig. 5), all clear characteristics of the choroid plexus. Finding such a high enrichment of choroid marker genes by comparing high vs low *TTR* as well as a similar analysis of *FOLR1* groups is highly unlikely due to chance and the most likely explanation is unequal distribution of choroid contamination.

## Discussion

Our findings suggest that the highly uneven distribution of *TTR* and other choroid marker gene expression is most likely due to inadvertent inclusion of choroid plexus during tissue dissection. These findings were present in multiple species. Tissues in closer proximity to the choroid plexus are more likely to have contamination, and are especially notable for the hippocampus, a structure of intense study given its role in memory formation and several diseases. Although these proximal structures were most prone to potential contamination, it is important to note that even a few samples from the frontal cortex in the GTEx dataset had *TTR* expression above the 90th percentile. It is impossible to prove that any individual experiment suffers from contamination. However, our differential expression analysis of “high” vs “low” *TTR* expressing samples from GTEx revealed a clear enrichment of other choroid marker genes and pathways (Fig. 3, Supplementary Table 4, and Supplementary Fig. 1) and the most reasonable explanation is unintended choroid contamination.

It is possible that choroid plexus contamination may not be limited to bulk transcriptome data. Single-cell RNAseq is becoming a widely popular tool for profiling thousands of individual cell types within a system for characterizing cell function, regulation, and interactions among cells [14, 15]. Several methods exist for isolating single cells including microfluidic and microwells [14, 15]. Once the cells are sorted, they are lysed to prepare for target library sequencing. Doublets and barcode swapping have been previously reported [16, 17], and we hypothesize that choroid plexus cells are no exception. Choroid plexus cells rapidly transiting the high-pressure microfluidics of the single-cell chip could lyse, resulting in *Ttr*, *Folr1*, and *Prlr* transcripts skewing or contaminating cell type analyses. Previous single-cell analysis of 20 mouse tissues revealed incorrect clustering of pancreatic endothelial cells in non-pancreas tissues due to contamination from pancreatic acinar genes [18, 19]. Choroid plexus contamination is also likely to occur with proteomics, lipidomics, metabolomics, and other “omics” datasets. However, for those datasets, other choroid markers (e.g., *FOLR1* or *PRLR*) are probably better suited to infer contamination since *TTR* is a highly secreted protein.

Recent publications have shown that the choroid plexus is a crucial target for COVID-19 [20] as well as a critical site for dysfunction in AD [21]. Yang et al. [20] show that 41 genes are upregulated and shared between COVID-19 and AD choroid plexus microglial clusters compared to control samples and suggests that COVID-19 and AD share a propensity for choroid plexus dysfunction [20]. Unfortunately, there is currently no systematic evaluation of choroid plexus contamination among brain banks from healthy and diseased donors. Investigators should examine the level of choroid plexus markers such as *TTR, FOLR1*, and *PRLR* to determine unintended group-level differences or unlikely high levels of expression for *TTR*, *FOLR1*, and *PRLR* before inferring biological interpretations when utilizing these data sets.

To avoid costly analyses that may lead to misinterpretation, we suggest that researchers might prescreen samples via RT-qPCR or western blot for choroid markers before sequencing or other omics studies. If utilizing already sequenced data, researchers should percentile rank choroid plexus markers for each sample and determine if there are unintended group-level differences, or biologically unreasonably high expression levels of choroid plexus markers. To avoid contaminated omics profiling and unintended missed biological conclusions, researchers should carefully consider the experimental design to prevent contamination and the biology of the sample to identify potentially contaminated samples. We highlight here an example of tissue profiling that results in unwanted contamination of the target region within the brain, but other tissues with poorly discerned tissue boundaries might also suffer from unintentional introduction of an adjacent structure that could cause similar problems and thus caveat emptor.

## Supplementary information

Supplementary information file

Supplementary Table 1

Supplementary Table 2

Supplementary Table 3

Supplementary Table 4

Supplementary Table 5

Supplementary Figure 1

Supplementary Figure 2

Supplementary Figure 3

Supplementary Figure 4

Supplementary Figure 5
