# Supplementary material for: Widespread choroid plexus contamination in sampling and profiling of brain tissue
Source: Mol Psychiatry. 2022 Jan 5;27(3):1839–47. doi: 10.1038/s41380-021-01416-3 (PMC9095494; doi:10.1038/s41380-021-01416-3)
Supplement: Supplementary file 1 — Supplementary information file [file 41380_2021_1416_MOESM1_ESM.docx]

### SUPPLEMENTAL

**Supplemental Table 1. GEO profiles and TTR/Ttr rank priority.** A one-way ANOVA test was run on each of the 158 profiles which assessed the *TTR/Ttr* rank across experimental groups. Each profile was assigned 1 of 5 priority numbers to categorize the level of *TTR/Ttr* contamination (Table 1). Fifteen of the GEO profiles produced NaN values during the ANOVA test and were removed from downstream analysis.

**Supplemental Table 2. Allen Brain hippocampus gene differential expression.** Differentially expressed genes in the “low” and “high” contamination hippocampus samples and repeated for female and male only samples.

**Supplemental Table 3. GTEx brain regions TTR expression.** The sample size for twelve GTEx brain regions. The mean and median transcripts per million (TPM) expression of *TTR*. The mean percentile rank of *TTR.*

**Supplemental Table 4. GTEx hippocampus gene differential expression.** Differentially expressed genes in the “low” and “high” contamination hippocampus samples and repeated for female and male only samples.

**Supplemental Table 5**. **DropViz.org search for *Ttr, Folr1, and Prlr* expression in brain regions.** We searched the expression of *Ttr*, *Folr1*, and *Prlr*, in the two cell populations neural stem cells and oligodendrocytes, and included a table obtained from querying dropViz.org for these genes for all brain regions that are noted by DropViz.org.

**Supplemental Figure 1. Metascape enrichment of GTEx hippocampus up-regulated genes.** Differential expression in GTEx hippocampus between likely not contaminated and potentially contaminated samples. Metascape analysis of 69 up-regulated genes in the potentially contaminated group shows enrichment for axoneme assembly and cilium movement.

**Supplemental Figure 2. Violin jitter of the log_2_(*TTR)* expression in TPM for each age bin. A)** Allen Brain Atlas donors range from 75 to 100+ years old and are denoted in bins every five years. **B)** GTEx donors range from 20 to 79 years old and are denoted in bins every ten years. Utilizing a non-parametric Kruskal-Wallis rank-sum, we determined differences in *TTR* expression distributions among the age bins and found no statistically significant difference between *TTR* expression by different age bins for the Allen Brain Atlas data (H = 87.66, df = 91, *p-value*= 0.58) or for the GTEx data (H = 196, df = 196, *p-value*= 0.49).

**Supplemental Figure 3. Gene differential expression between high and low contamination Allen Brain Atlas hippocampus for genetic female and genetic male samples independently. A)** Histogram of the log_2_(*TTR)* expression among hippocampus samples within the Allen Brain Atlas data for female donors and **B)** male donors. The blue left shaded regions indicate the samples that likely do not have choroid plexus contamination, log_2_(*TTR*) < 3.32. The right red shaded region indicates samples that potentially have choroid plexus contamination, log_2_(*TTR*) > 5.32. **C)** Volcano plot showing the gene differential expression between the potentially contaminated and likely not contaminated Allen Brain Atlas hippocampus female samples (n = 28) and **D)** male hippocampus samples (n = 40). Vertical lines show the log_2_ fold change of 1 and -1. Horizontal line indicates the adjusted *p*-value cutoff of 0.05. Each point represents a gene, points in blue are down-regulated, FDRq < 0.05 and the log_2_ fold change less than -1. Up-regulated genes are indicated in red, FDRq < 0.05 and the log_2_ fold change greater than 1. Genes that are not differentially expressed are shown in gray.

**Supplemental Figure 4. Gene differential expression between high and low contamination GTEx hippocampus for genetic female and genetic male samples independently. A)** Histogram of the log_2_(*TTR)* expression among hippocampus samples within the GTEx data for female donors and **B)** male donors. The blue left shaded regions indicate the samples that likely do not have choroid plexus contamination, log_2_(*TTR*) < 3.32. The right red shaded region indicates samples that potentially have choroid plexus contamination, log_2_(*TTR*) > 5.32. **C)** Volcano plot showing the gene differential expression between the potentially contaminated and likely not contaminated GTEx hippocampus female samples (n = 43) and **D)** male hippocampus samples (n = 91). Vertical lines show the log_2_ fold change of 1 and -1. Horizontal line indicates the adjusted *p*-value cutoff of 0.05. Each point represents a gene, points in blue are down-regulated, FDRq < 0.05 and the log_2_ fold change less than -1. Up-regulated genes are indicated in red, FDRq < 0.05 and the log_2_ fold change greater than 1. Genes that are not differentially expressed are shown in gray.

**Supplemental Figure 5. Metascape enrichment of GTEx hippocampus up-regulated genes between high and low contamination samples with FOLR1 as the marker.** Differential expression in GTEx hippocampus between likely not contaminated and potentially contaminated samples. Metascape analysis of 178 up-regulated genes in the potentially contaminated group shows enrichment for cilium movement and axonemal dynein complex assembly.
