# Supplementary figures and images for: Widespread choroid plexus contamination in sampling and profiling of brain tissue
Source: Mol Psychiatry. 2022 Jan 5;27(3):1839–47. doi: 10.1038/s41380-021-01416-3 (PMC9095494; doi:10.1038/s41380-021-01416-3)

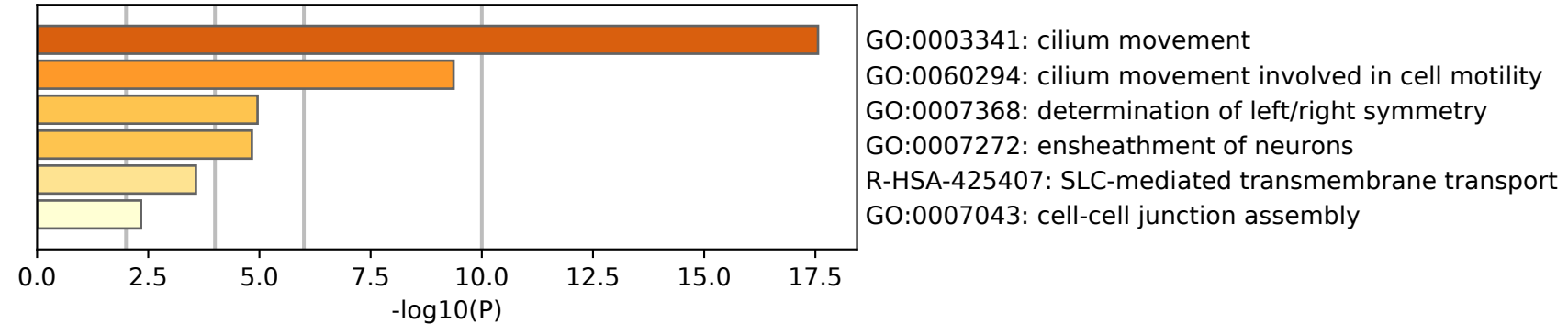

Supplement: Supplementary file 7 — Supplementary Figure 1 [file 41380_2021_1416_MOESM7_ESM.pdf]

**A)** Allen Brain Atlas

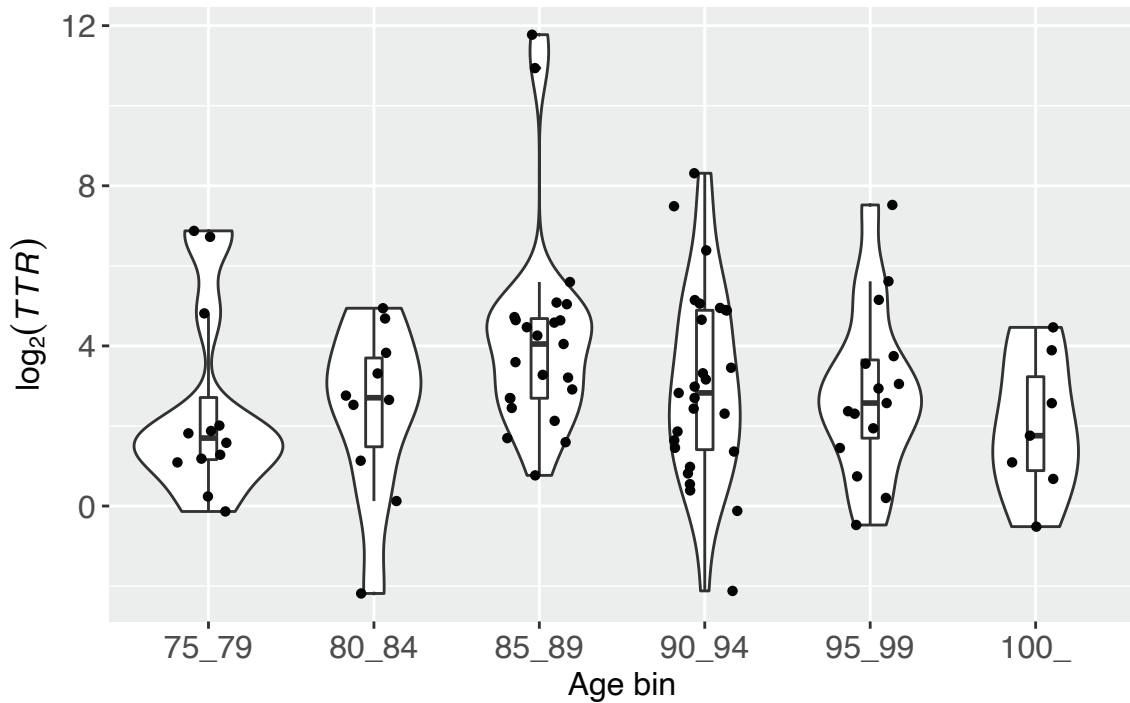

**B)** GTEx

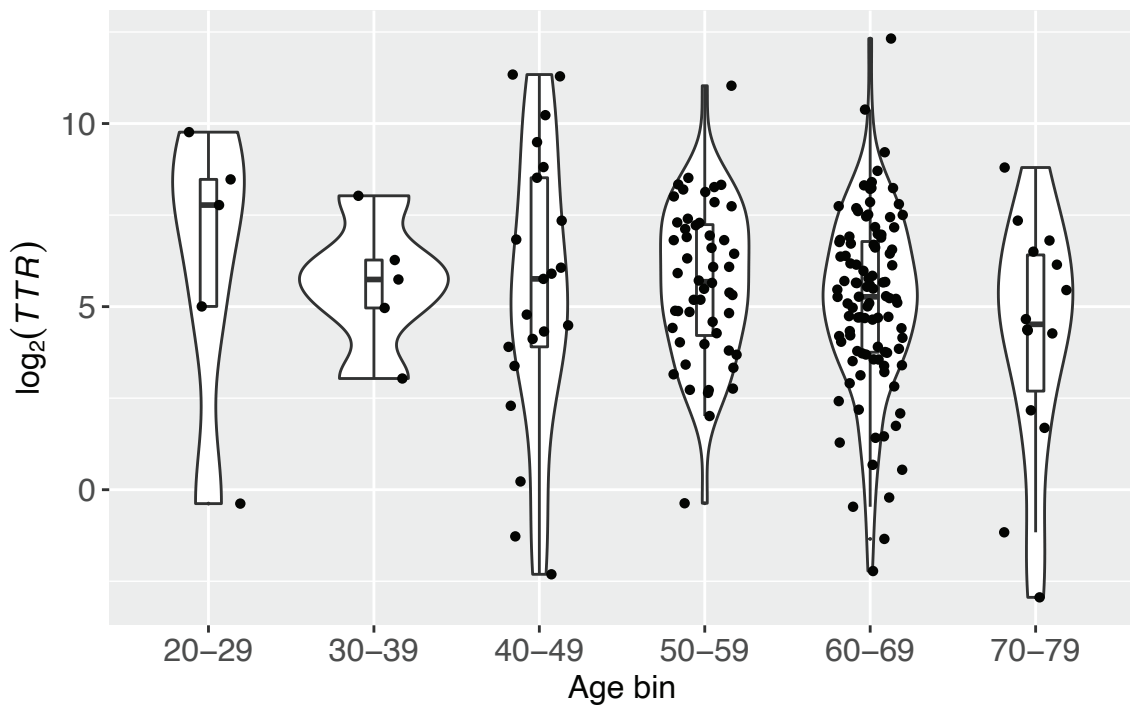

Supplement: Supplementary file 8 — Supplementary Figure 2 [file 41380_2021_1416_MOESM8_ESM.pdf]

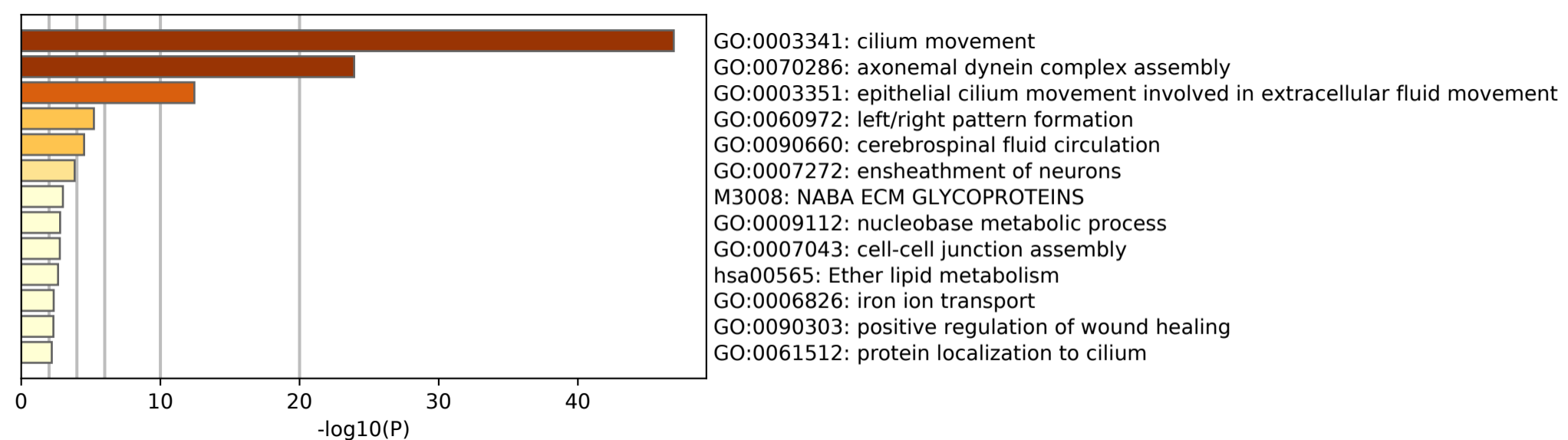

Supplement: Supplementary file 11 — Supplementary Figure 5 [file 41380_2021_1416_MOESM11_ESM.pdf]
