# Supplementary material for: Widespread choroid plexus contamination in sampling and profiling of brain tissue
Source: Mol Psychiatry. 2022 Jan 5;27(3):1839–47. doi: 10.1038/s41380-021-01416-3 (PMC9095494; doi:10.1038/s41380-021-01416-3)
Supplement: Supplementary file 9 — Supplementary Figure 3 [file 41380_2021_1416_MOESM9_ESM.pdf]

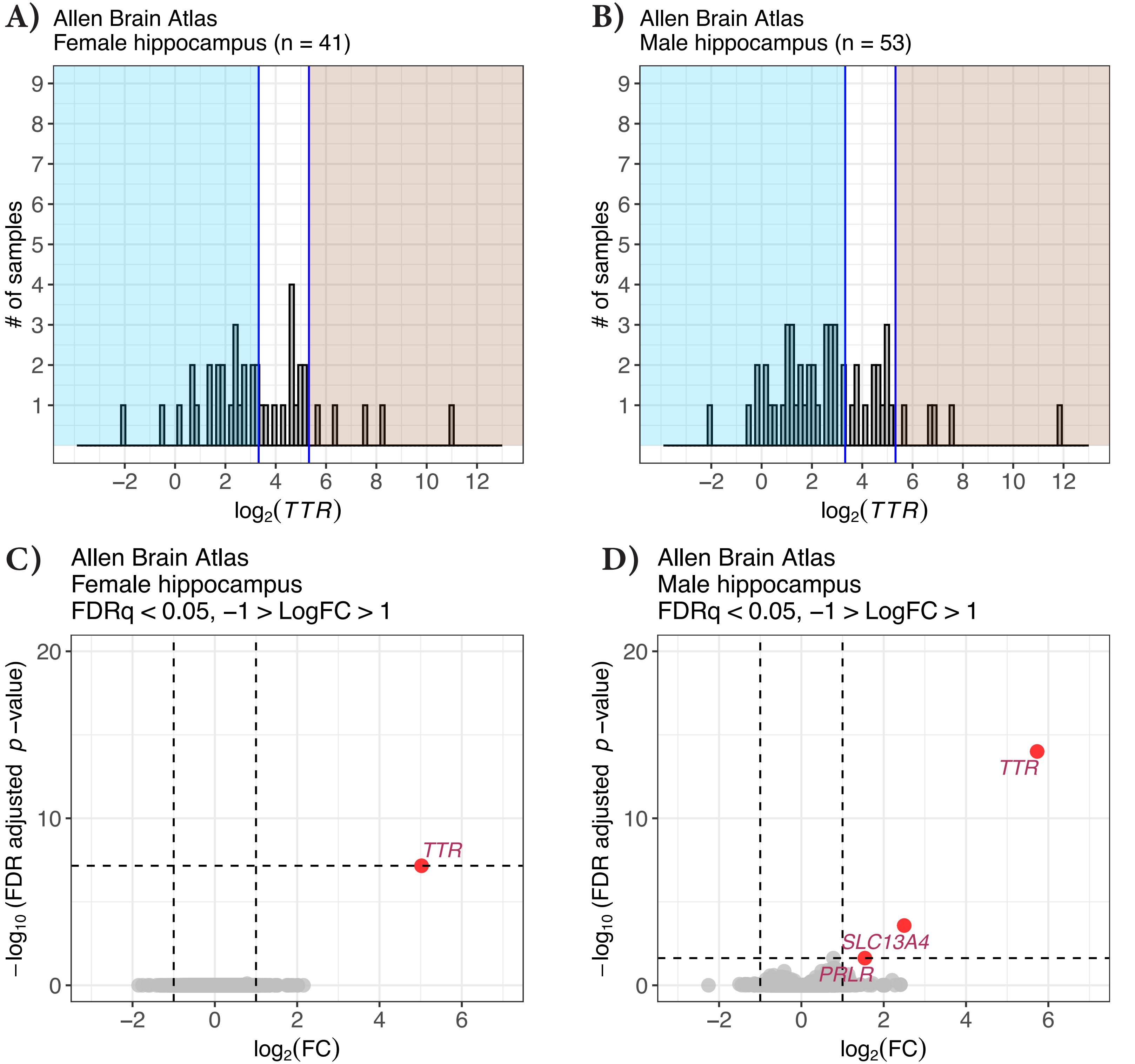
A)
Allen Brain Atlas
Female hippocampus (n = 41)
# of samples
log2(TTR)
B)
Allen Brain Atlas
Male hippocampus (n = 53)
# of samples
log2(TTR)
C)
Allen Brain Atlas
Female hippocampus
FDRq < 0.05, −1 > LogFC > 1
−log10 (FDR adjusted p −value)
TTR
log2(FC)
D)
Allen Brain Atlas
Male hippocampus
FDRq < 0.05, −1 > LogFC > 1
−log10 (FDR adjusted p −value)
TTR
SLC13A4
PRLR
log2(FC)
