# Supplementary material for: Widespread choroid plexus contamination in sampling and profiling of brain tissue
Source: Mol Psychiatry. 2022 Jan 5;27(3):1839–47. doi: 10.1038/s41380-021-01416-3 (PMC9095494; doi:10.1038/s41380-021-01416-3)
Supplement: Supplementary file 10 — Supplementary Figure 4 [file 41380_2021_1416_MOESM10_ESM.pdf]

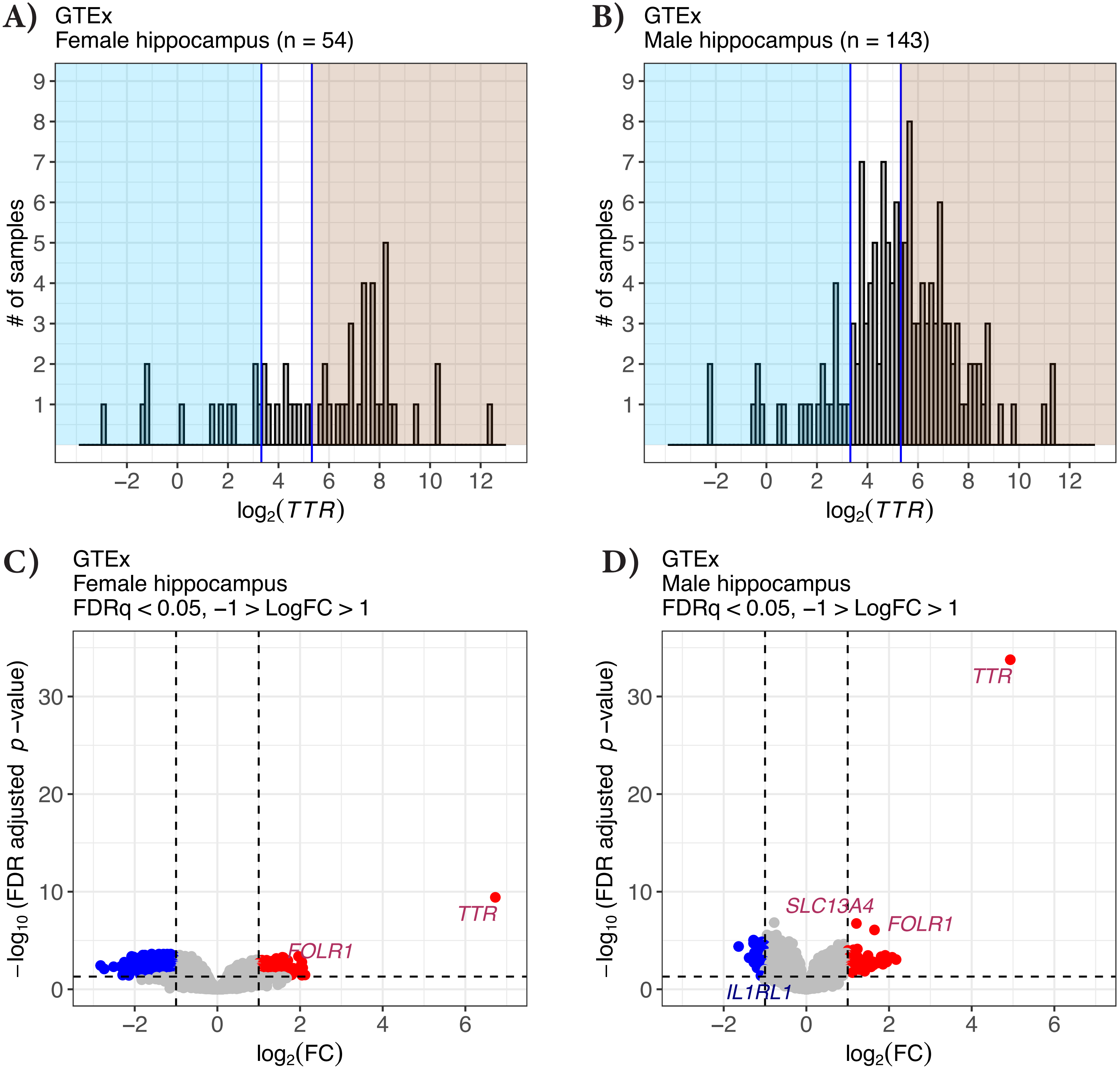

A)
GTEx
Female hippocampus (n = 54)
# of samples
log2(TTR)
B)
GTEx
Male hippocampus (n = 143)
# of samples
log2(TTR)
C)
GTEx
Female hippocampus
FDRq < 0.05, −1 > LogFC > 1
−log10 (FDR adjusted p −value)
log2(FC)
TTR
FOLR1
D)
GTEx
Male hippocampus
FDRq < 0.05, −1 > LogFC > 1
−log10 (FDR adjusted p −value)
log2(FC)
TTR
SLC13A4
FOLR1
IL1RL1
